# Right Atrial Mural Thrombus: A Rare Case Complicated by Superior Vena Cava Obstruction

**DOI:** 10.7759/cureus.102495

**Published:** 2026-01-28

**Authors:** Dilkash Harryprasadh, Zeyn Mahomed, Shiraz Harypursat, Peter Beskyd, Craig Beringer

**Affiliations:** 1 Internal Medicine, Chris Hani Baragwanath Academic Hospital, Johannesburg, ZAF; 2 Emergency Medicine, University of the Witwatersrand, Johannesburg, Johannesburg, ZAF; 3 Emergency Department, Chris Hani Baragwanath Academic Hospital, Johannesburg, ZAF

**Keywords:** human immuno deficiency virus, right atrial thrombus, superior vena cava (svc) obstruction, systemic thrombolysis, transthoracic echocardiogram (tte)

## Abstract

Right heart thrombi are rare but potentially life-threatening, often arising from deep vein thrombosis or structural heart disease. They are strongly associated with pulmonary embolism, and their diagnosis is frequently delayed, leading to high morbidity and mortality. Bedside ultrasound has emerged as a critical tool for the rapid identification of right heart thrombi, enabling timely intervention.

The authors present a case of a right atrial thrombus in an immunocompromised adult male patient. In this case report, the authors describe the investigations that led to the rapid identification of the right heart thrombus. Therapeutic options traditionally include surgical embolectomy, thrombolysis, anticoagulation, and percutaneous retrieval techniques. The authors were based in a resource-limited setting and will discuss the decision-making process that ensued.

## Introduction

Intracardiac thrombi are potentially life-threatening entities, with those located in the right atrium (right atrial thrombi, or RAT) carrying a particularly high risk of morbidity and mortality due to the threat of pulmonary embolism. RAT is most commonly associated with indwelling central venous catheters, pacemaker leads, atrial fibrillation, and heart failure. Complications range from pulmonary embolism and septic emboli to, rarely, obstruction of right-sided cardiac flow [1].

RAT are frequently categorized by echocardiographic appearance: type A thrombi are serpentine, highly mobile, and often associated with indwelling catheters; type B thrombi are more broadly based, less mobile, and frequently attached to the atrial wall or interatrial septum. Among these, type B thrombi are notably less common and are associated with a reported in-hospital mortality rate as high as 30-40%, yet they remain under-represented in the case report literature [1].

This report details a rare and clinically critical presentation of a large, type B right atrial thrombus that led to a consequential secondary complication: functional superior vena cava (SVC) obstruction. While the etiology and management of RAT have been discussed, cases documenting SVC obstruction as a direct complication are scarce. We present this case to highlight the diagnostic challenges, unique hemodynamic consequences, and multidisciplinary management required when a type B RAT precipitates SVC syndrome, underscoring a noteworthy and severe manifestation of this already high-risk condition.

## Case presentation

An African male in his fifties presented to the emergency department (ED) with a three-month history of worsening exertional dyspnoea (Modified Medical Research Council Grade 3), generalized swelling of his body, and facial swelling that was worse in the morning. He also reported an associated intermittent, non-productive cough. No chest pain, fever, weight loss, or night sweats were reported. The patient is HIV-positive and was on first-line fixed-dose combination antiretroviral therapy. Regarding social history, the patient admitted to significant ethanol use and is a long-standing smoker of tobacco with a 20-pack-year history.

Physical examination revealed central cyanosis with hypoxemia on room air (pulse oximetry of 70% on room air, increasing to 94% on a non-rebreather mask). He was noted to have anasarca with facial swelling and conjunctival plethora. His cardiovascular examination was significant for a raised jugular venous pulse, displaced apex beat, a parasternal heave, and a palpable pulmonary valve impulse. He had a wide splitting of the S2 heart sound. In the normal heart, the second sound (S2) is usually single during expiration. During inspiration, S2 is made of the aortic valve closure (A2), which happens first, and the pulmonic valve closure, which happens second. Other notable findings included significant ascites.

The patient's electrocardiogram revealed a sinus rhythm at 96 beats per minute with a right axis deviation, dominant R wave in leads V1 and aVR, and poor precordial R wave progression. His venous blood gas revealed a mixed respiratory and metabolic acidosis with a pH of 7.21, pCO2 of 55, and HCO3 of 17, with a lactate of 4.2.

**Figure 1:**
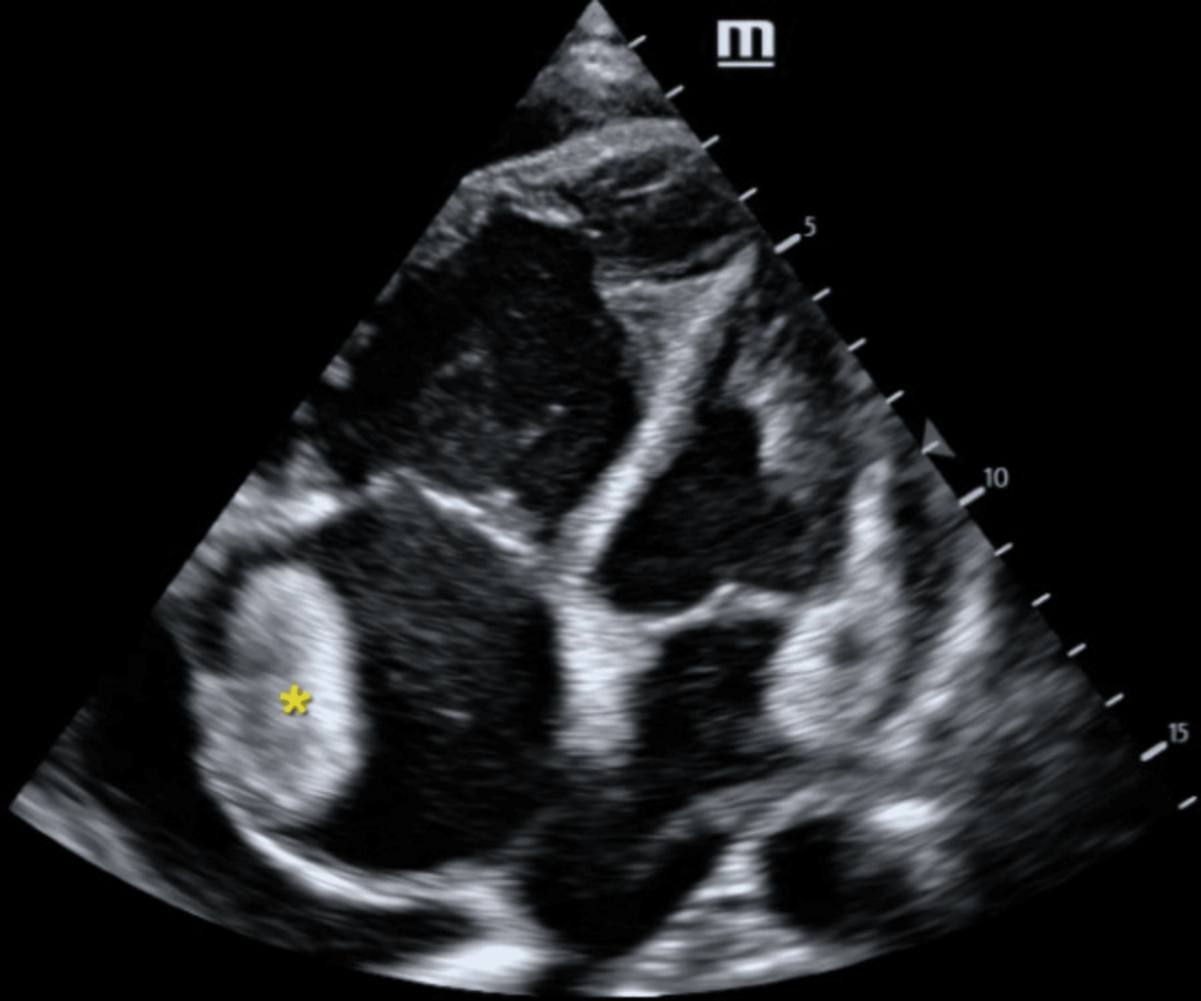
Apical four chamber view demonstrating an isoechoic mass attached to the lateral right atrial wall (yellow asterisk). Right ventricular enlargement is evident.

Emergency point-of-care ultrasound was performed by an accredited practitioner. Echocardiography revealed a significantly dilated right ventricle and atrium with flattening and left-sided deviation of the interventricular septum, along with poor contractility of both the right ventricle and atrium. No overt regional wall motion abnormalities were noted. Of interest was an isoechoic mass that appeared attached to the lateral right atrial wall, measuring 35 x 44 mm (Figure 1). His inferior vena cava was dilated and non-variable with inspiration. Other findings of note included pulmonary regurgitation and tricuspid regurgitation (tricuspid regurgitation gradient= 24 mmHg); however, his left ventricular ejection fraction appeared grossly preserved.

**Figure 2:**
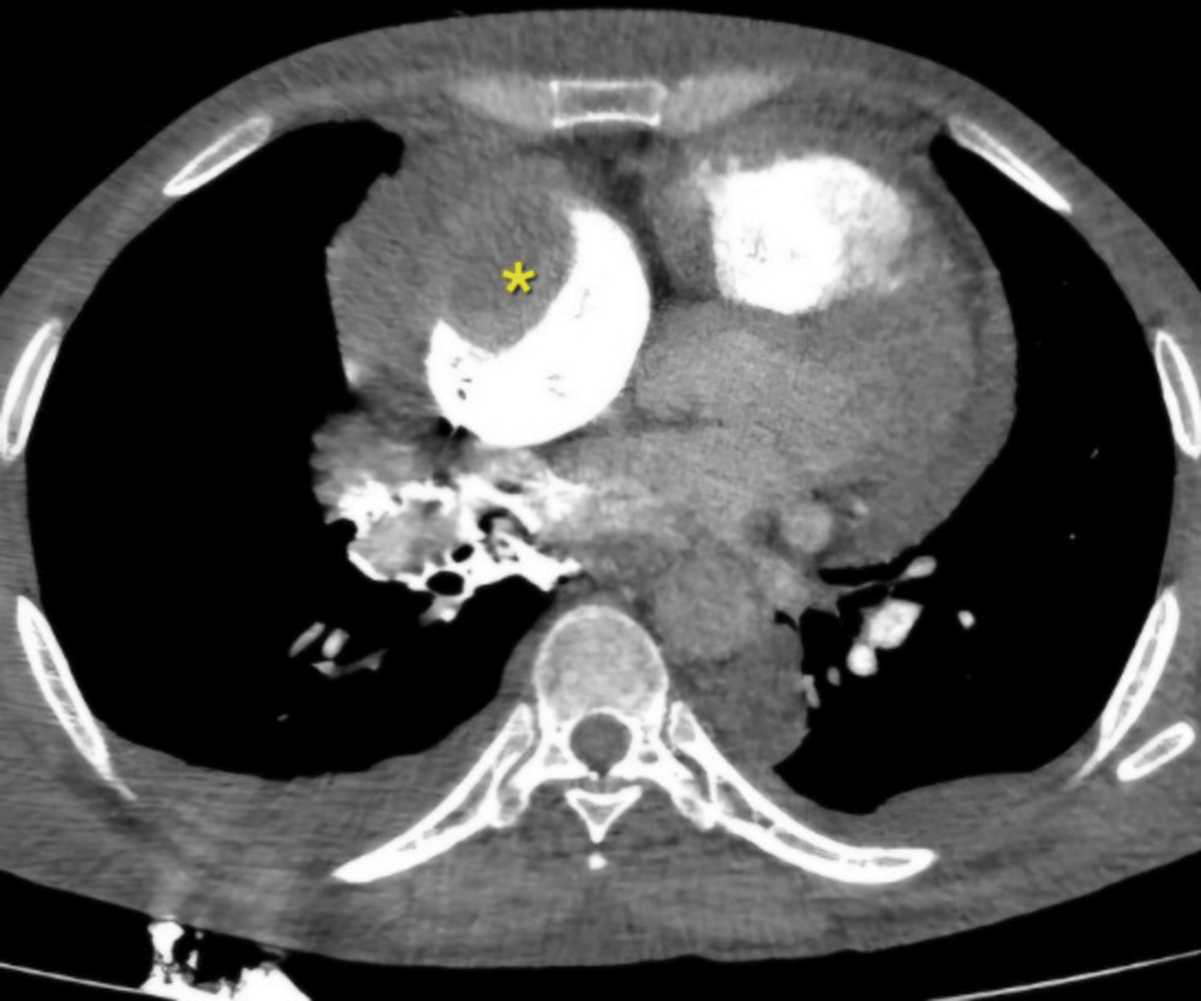
CT chest demonstrating right atrial thrombus (yellow asterisk)

**Figure 3:**
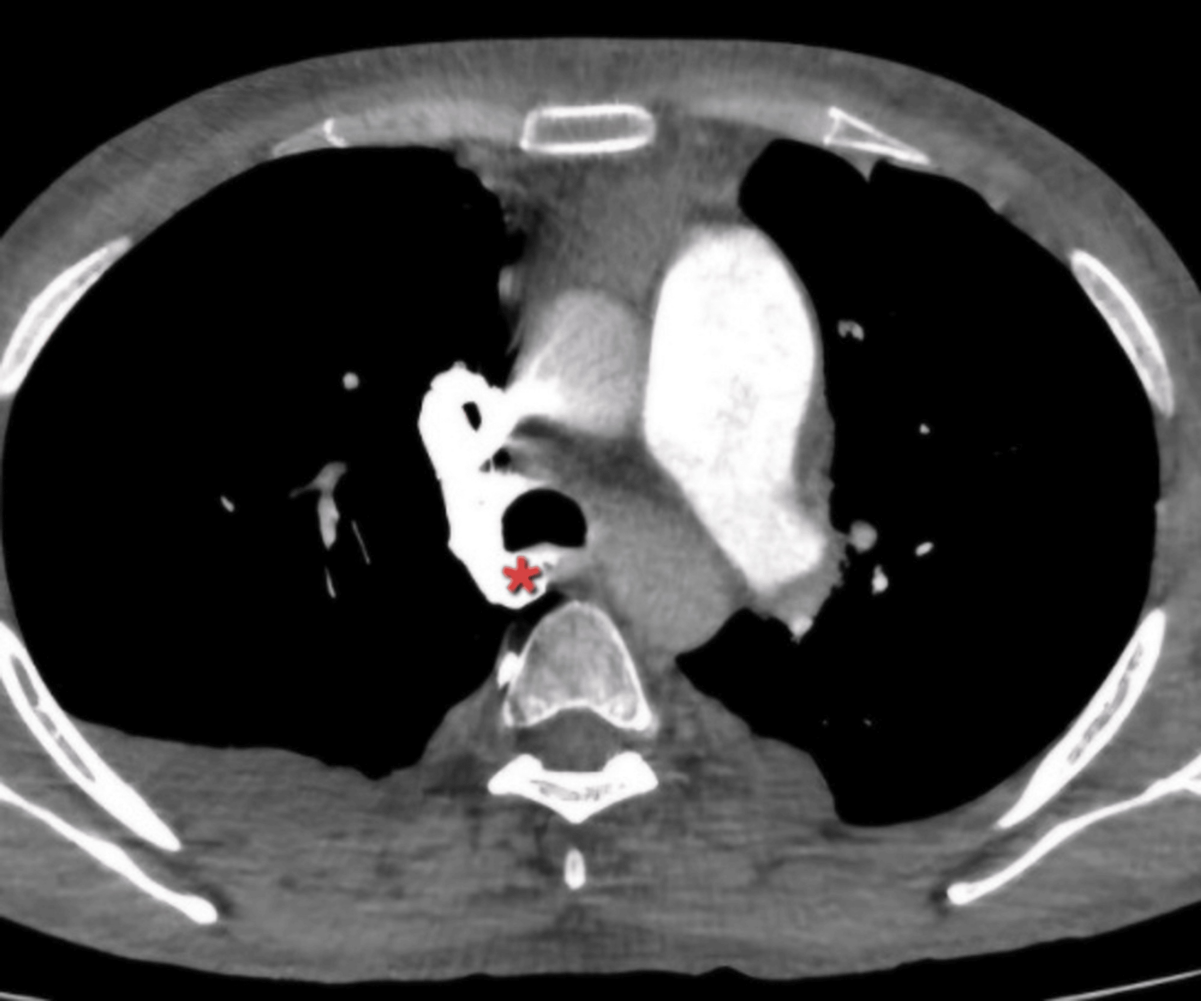
CT chest demonstrating dilated azygos vein measuring 12.1mm (red asterisk)

A computed tomography pulmonary angiogram was performed, which showed a hypodense, well-defined lesion in the right atrium, likely a thrombus (Figure 2). It measured 55 x 43 x 33 mm. The difference in measurements between the point-of-care ultrasound and CT could reflect the inherent differences between these modalities between the SVC obstruction was evidenced by a dilated azygous vein and other smaller mediastinal veins (Figure 3). There were features consistent with pulmonary hypertension, but no pulmonary embolus was present. The lungs were noted to have emphysematous changes.

Following the above investigations, in consultation with the specialist emergency physician on call, a decision was made not to administer thrombolytics to the patient. The rationale for this decision was due to the chronicity of the right atrial thrombus and the hemodynamic stability of the patient. Anticoagulation with low molecular weight heparin at therapeutic doses was initiated, and diuresis was achieved with a loop diuretic. Multidisciplinary team management was initiated in conjunction with the internal medicine team, and the patient was subsequently admitted to the medical wards.

Whilst in the wards, the patient was managed with a combination of fluid restriction, daily weights, diuresis, and diagnostic and therapeutic ascitic taps as symptomatic management of his right heart failure. Ascitic fluid was confirmed to be transudative in nature. In view of the RAT and SVC obstruction, the patient was anticoagulated initially with therapeutic doses of low molecular weight heparin, subsequently changed over to rivaroxaban, a direct oral anticoagulant, requiring minimal ongoing monitoring of its therapeutic effect.

Ongoing inpatient investigation included workup of his prothrombotic state, the results revealing no significant abnormality. Further inpatient imaging included a formal abdominal ultrasound, which confirmed the presence of massive, simple ascites with features of portal hypertension and an ultrasonographically normal liver and kidneys. An inpatient formal echocardiogram revealed similar findings to those illustrated in the ED.

The patient responded well to the above measures, evidenced by both patient-reported and clinician-observed clinical improvement. Repeat bedside focused cardiac ultrasonography revealed a persistent right atrial thrombus, unchanged in size following two weeks of anticoagulation.

## Discussion

Right atrial thrombi (RAT) can be classified into three morphologically distinct types, according to a 1989 report by the European Working Group on Echocardiography [1]. The present case report focuses on type B RAT, which is described as immobile clots attached to the right atrial free wall in association with underlying structural heart disease or intracardiac foreign bodies. The prevalence of RAT (particularly type B RAT) is poorly defined in the current literature. Type B RAT appears to have a better prognosis and fewer adverse outcomes, despite a similar incidence of pulmonary embolism when compared to the other two types of RATs [1].

Superior vena cava (SVC) syndrome refers to a constellation of symptoms associated with obstruction of the SVC. Common causes include a range of non-benign pathologies; however, benign causes are of increasing significance [2,3]. The presentation of SVC obstruction and its severity depend on both the development of collateral circulation, which itself depends on the level and rapidity of development of obstruction [3]. Collateral venous flow develops primarily through the azygous-hemiazygous veins, intercostal and lumbar veins, while a variety of other lesser collaterals also exist [4]. Symptoms of SVC obstruction include facial and conjunctival edema and plethora, proptosis, dyspnea, headache, papilledema, and a reduced level of consciousness [3].

## Conclusions

The present case demonstrates a rare presentation of a right atrial mural thrombus causing SVC obstruction with collateral formation, a combination poorly described in the current literature. While some cases have documented this occurring in patients with indwelling venous catheters or pacing cables, this case is unique in that the patient developed a right atrial thrombus spontaneously with no overt underlying structural heart disease or foreign body. His RAT was managed conservatively with anticoagulation alone, and his other existing and newly diagnosed comorbidities were managed in a multidisciplinary team setting.

## References

[1] Kronik G (1989). The European Cooperative Study on the clinical significance of right heart thrombi. Eur Heart J.

[2] Seligson MT, Surowiec SM (2024). Superior Vena Cava Syndrome. https://www.ncbi.nlm.nih.gov/books/NBK441981/.

[3] Friedman T, Quencer KB, Kishore SA, Winokur RS, Madoff DC (2017). Malignant venous obstruction: superior vena cava syndrome and beyond. Semin Intervent Radiol.

[4] Kapur S, Paik E, Rezaei A, Vu DN (2010). Where there is blood, there is a way: unusual collateral vessels in superior and inferior vena cava obstruction. Radiographics.

